# Extremely distinct microbial communities in closely related leafhopper subfamilies: Typhlocybinae and Eurymelinae (Cicadellidae, Hemiptera)

**DOI:** 10.1128/msystems.00603-25

**Published:** 2025-06-26

**Authors:** Michał Kobiałka, Dariusz Świerczewski, Marcin Walczak, Weronika Urbańczyk

**Affiliations:** 1 Laboratory of Experimental Neuropathology, Institute of Zoology and Biomedical Research, Faculty of Biology, Jagiellonian University 49589, Kraków, Poland; 2 Faculty of Mathematics and Natural Sciences, Jan Długosz University, Częstochowa, Poland; 3 Institute of Biology, Biotechnology and Environmental Protection, Faculty of Natural Sciences, University of Silesia in Katowice, Katowice, Poland; The Pennsylvania State University, State College, Pennsylvania, USA

**Keywords:** endosymbionts, symbiotic bacteria, symbiotic fungi, leafhoppers, insect microbiome, microbial ecology

## Abstract

Among the Hemiptera insects, a widespread way of feeding is sucking sap from host plants. Due to their nutrient-poor diet, these insects enter into obligate symbiosis with their microorganisms involved in the synthesis of components essential for host survival. However, within the Cicadellidae family, there is a relatively large group of mesophyll feeders—Typhlocybinae—that is considered to be devoid of obligate symbiotic companions. In this work, we examine the composition of microorganisms in this subfamily and compare the results with their close relatives—the Eurymelinae subfamily. To study the microbiome, we used high-throughput next-generation sequencing (NGS, Illumina) and advanced microscopic techniques, such as transmission electron microscopy (TEM) and fluorescence *in situ* hybridization (FISH), in a confocal microscope. In the bodies of Typhlocybinae insects, we did not detect the presence of microorganisms deemed to be obligate symbionts. Their microbial communities consist of facultative symbionts, mainly alphaproteobacteria such as *Wolbachia* or *Rickettsia* as well as others that can be considered as facultative, including *Spiroplasma*, *Acidocella*, *Arsenophonus*, *Sodalis*, *Lariskella*, *Serratia*, *Cardinium*, and *Asaia*. On the other hand, the Eurymelinae group is characterized by a high diversity of microbial communities, both obligate and facultative, similar to other Cicadomorpha. We find co-symbionts involved in the synthesis of essential amino acids such as *Karelsulcia*, betaproteobacteria *Nasuia,* or gammaproteobacteria *Sodalis*. In other representatives, we observed symbiotic yeast-like fungi from the family Ophiocordycipitaceae or *Arsenophonus* bacteria inhabiting the interior of *Karelsulcia* bacteria. Additionally, we investigated some aspects of symbiont transmission and the phylogeny of symbiotic organisms and their hosts.

**IMPORTANCE:**

The Typhlocybinae and Eurymelinae leafhoppers differ significantly in their symbiotic communities. They have different diets, as Typhlocybinae insects feed on parenchyma, which is richer in nutrients, while Eurymelinae, like most representatives of Auchenorrhyncha, consume sap from the phloem fibers of plants. Our work presents comprehensive studies of 42 species belonging to the two above-mentioned, and so far poorly known, Cicadomorpha subfamilies. Phylogenetic studies indicate that the insects from the studied groups have a common ancestor. The diet shift in the Typhlocybinae leafhoppers contributed to major changes in the composition of microorganisms inhabiting the body of these insects. Research on the impact of diet on the microbiome and the subsequent consequences on the evolution and adaptation of organisms plays an important role in the era of climate change.

## INTRODUCTION

The Typhlocybinae and Eurymelinae are closely related insect subfamilies, belonging to the Cicadellidae, known as leafhoppers—the largest family of Cicadomorpha, which, collectively with Fulgoromorpha, are placed within Hemiptera (1, 2). Moreover, the Typhlocybinae is the second largest subfamily of the family Cicadellidae (3–5); however, recent samplings of tropical faunas indicate that most species of Typhlocybinae remain undescribed, so this taxon can be far larger than any other leafhopper subfamily (4). This monophyletic group of hemipterans includes many plant pests and invasive species that are rapidly spreading across the globe; thus, it is claimed that they may be ideal markers of climate change (6–10). The Typhlocybinae group is divided into seven tribes, and even in the European fauna, all currently distinguished tribes of this group have their representatives. The Eurymelinae is the most closely related subfamily to the Typhlocybinae (2). This subfamily is regarded as a paraphyletic group of arboreal leafhoppers, which comprises monophyletic groups, formerly treated as subfamilies, i.a., Eurymelini, Idiocerini, and Macropsini (11). The research by Xue and coworkers (11) showed that the Eurymelinae includes 11 tribes, and there are members of two tribes—Idiocerini and Macropsini—present in Europe.

Although the leafhoppers from the subfamilies mentioned above have a lot in common and often occupy similar ecological niches, they differ slightly in their feeding mode. The Typhlocybinae are mostly arboreal species, preferentially feeding on the parenchyma of the leaf of their host plants (9, 12). Insect pierces the cuticle and epidermis of the leaf blade using piercing and sucking mouthparts and then sucks up the content of the mesophyll cells. The species belonging to the Eurymelinae are mostly monophagous forms that feed mainly on trees or shrubs. Although they often share a common habitat with Typhlocybinae leafhoppers, they are exclusively phloem feeders.

Since most hemipterans feed on phloem or xylem sap of plants, which are deficient in essential nutrients, they host obligate symbiotic microorganisms—bacteria or fungi (termed yeast-like symbionts). The symbionts of hemipterans are traditionally divided into two groups: obligate symbionts (also termed primary symbionts) and facultative symbionts (known also as secondary symbionts) (13–16). Primary symbionts occur in all the members of particular taxa because they infected the ancestor of this group of insects. Facultative symbionts are younger insect associates. Therefore, they are often present only in some populations. Obligate symbionts supply their host with nutrients missing from their diet: essential amino acids, cofactors, and vitamins (14, 17, 18). Since symbiotic bacteria have strongly reduced genomes, they cannot be cultivated outside the body of the host insect on laboratory media (19–21). The obligate symbionts are harbored in specialized cells of host insects called bacteriocytes (if bacteria) or mycetocytes (if fungal symbionts), which are grouped into large, paired, distinctively colored structures termed bacteriomes or mycetomes. These microorganisms are transovarially (vertically) transmitted between generations. Facultative symbionts may occur both in bacteriocytes and in other types of insect cells (e.g., in fat body cells) or extracellularly (e.g., in hemolymph). Their function in many cases remains unclear. They may protect the host from heat stress, fungal pathogens, and parasitic hymenopterans (22–27).

In Cicadomorpha and Fulgoromorpha, several types of obligate symbionts are responsible for the synthesis of essential amino acids; therefore, they are called “coprimary symbionts” (18, 28–30). Most cicadomorphans and fulgoromorphans host ancient symbionts: the bacterium *Candidatus Karelsulcia muelleri* (phylum Bacteroidetes) and a betaproteobacterial symbiont. During further evolution, in some lineages of Auchenorrhyncha, additional microorganisms have been acquired, or the betaproteobacterium was replaced by other bacteria or yeast-like fungal symbionts (28, 31, 32). Nowadays, most cicadomorphans and fulgoromorphans host ancient symbionts: bacterium *Karelsulcia* and a betaproteobacterial symbiont, e.g., *Nasuia*, *Zinderia*, and *Vidania* (29–32).

There are few data on symbiotic associations in members of the Typhlocybinae group. It is hypothesized that these leafhoppers are the only auchenorrhynchans that do not harbor obligate symbionts due to the fact that they feed on the parenchyma of leaves (15, 33). So far, bacteriomes containing bacteria have not been found in representatives of this group. No evidence of transovarial transport of these microorganisms has been reported. To date, some facultative bacteria have been detected in the bodies of Typhlocybinae insects. Molecular studies indicate that some species are infected with *Wolbachia* or *Rickettsia*, and these infections potentially spread among populations through horizontal transmission (9, 34–37).

It is well known that symbiosis plays an important role in the ecology and evolution of both partners, symbiont and host-insect. Many studies prove that the distribution and composition of facultative symbionts may be dependent on biotic and abiotic factors (38, 39). Moreover, symbionts are considered to play a significant role in the diversification and expansion of the ecological niche of their host (40, 41). Results of recent studies suggest that microbial symbionts may depend on their host’s response toward abiotic stressors, such as changes in temperature or humidity, by expanding or constraining abiotic niche space (42–44). Because of genome reduction, many obligate symbionts are unable to cope with temperature changes and are considered their hosts’ “Achilles’ heels” in the context of temperature stress (21, 42, 43, 45). In the era of rapid environmental changes, understanding these relations seems to be crucial for the protection of biodiversity.

In this work, we verified the hypothesis that the Typhlocybinae is a group of leafhoppers that harbors no obligate symbionts, and then, we examined the symbiotic systems of their relatives—Eurymelinae leafhoppers. We carried out complex research on the symbiotic communities of the members of different tribes: taxonomic composition, occurrence, morphology, localization in the insect body, vertical transmission, and phylogeny. By combining morphological and molecular-based approaches (TEM, FISH, Illumina amplicon sequencing), we addressed the following three goals: (i) compare the composition of symbiotic communities of 42 species belonging to two studied subfamilies; (ii) evaluate whether examined insects exhibit convergent evolution with their symbionts; and (iii) assess the role of insect phylogeny and diet on the composition of symbiotic microorganisms.

## MATERIALS AND METHODS

### Molecular identification and phylogenetic analyses

In this study, we examined 158 individuals belonging to 42 species (18 of Eurymelinae and 24 of Typhlocybinae), taken from several localities in southern Poland (Table S1). Genomic DNA was extracted from specimens (fixed in 100% ethanol) using the GeneMATRIX Bio-Trace DNA Purification Kit (EURx), following the manufacturer’s protocol. Symbiotic microorganisms were determined based on their 16S rDNA (bacteria) and ITS1, ITS2 (fungal symbionts) sequences (46). Genomic DNA isolation was confirmed by amplifying the 16S rDNA and 18S rDNA using a pair of primers: V3–V4 (F357, R805) and NS1, FS2 (Table S2). The samples were sequenced by SEQme s.r.o. (Czech Republic) using the Illumina NovaSeq 6000 SF Flow Cell. The reads were classified into operational taxonomic units (OTUs) using Usearch and using the clustering method at a similarity threshold of 97% sequence identity. Then, the OTUs were assigned to the genus level based on databases (Silva 138.1, Unite 9, NCBI Genbank). Indices for alpha diversity were obtained using the Phyloseq package in R Studio. To perform the PCA analysis and create plots, we used the Factoextra package in R Studio (variables with low Cohen’s F coefficient [effect size <0.3] and outliers were discarded). The DNA sequences of the host molecular marker—mitochondrial cytochrome C oxidase I gene (COI)—were amplified using specific primers (Table S2) and then sequenced using the Sanger method (Genomed, Poland).

To determine if microbial community abundance was significantly associated with the most common diet plants, we implemented a series of different statistical tests. First, we calculated the Bray-Curtis dissimilarity matrix from microbiome abundance using the function vegdist from the Vegan R package and tested statistical significance using the adonis2 function (PERMANOVA) from the same package (47). As a fixed effect, we used a plant group (analogous to those used in PCA) or a plant genus affiliation. To control for the differences in microbiome composition resulting from phylogenetic relationships of studied insects, we included different levels of insect taxonomic level affiliation (insect subfamily or genus) in the restricted permutation design. We also use the glmmTMB package to fit generalized linear mixed models with Poisson family. We used plant group as a fixed effect and insect phylogenetic affiliation as a random effect (insect subfamily, genus, or taxa) or sample ID (either separately or with “genus” or “taxa” nested in “subfamily”). We used ANOVA with 999 permutations to test for model significance. We estimated the amount of variance in microbiome composition explained by insect diet using distance-based (Bray-Curtis) redundancy analysis (dbRDA), performed with the dbrda function in the Vegan package, using insect taxonomical affiliation (analogously to blocks used in restricted permutation design for PERMANOVA) as a “Condition” to provide correct restricted permutation tests.

The phylogenetic analyses were conducted using MrBayes software (48). For the Bayesian inference, four incrementally metropolis-coupled MCMC chains (three heated and one cold) were run for five million generations with trees sampled every 1000th generation. We used the GTR substitution model with gamma-distributed rate variation across sites and a proportion of invariable sites (“GTR+I+Γ”).

### Fluorescence *in situ* hybridization (FISH) and transmission electron microscopy (TEM)

For the FISH method, the adult females preserved in 90% ethanol were rehydrated, fixed in 4% paraformaldehyde for two hours at room temperature, and dehydrated through incubations in ethanol and acetone. For whole-mount FISH, the entire insects were bleached in 6% hydrogen peroxide in 80% ethanol for two weeks to quench autofluorescence. For the resin section FISH, the material was embedded in Technovit 8100 resin and cut into sections (1 µm thick) using an ultramicrotome Leica EMUC7. The slides or the whole samples were incubated in hybridization buffer and probes (Table S2) overnight at room temperature (49). The hybridized samples were examined using a confocal laser scanning microscope Zeiss Axio Observer LSM 710 and ZEN (Zeiss) software. The experiments were repeated on three different individuals from each species, for a total of 126 individuals.

For TEM specimens, material was preserved using 2.5% glutaraldehyde in phosphate buffer. The fixed material was stored at a temperature of 4°C and then postfixed in 1% osmium tetroxide for 1.5 hours. Finally, after being dehydrated in a graded series of ethanol and acetone, the material was embedded in epoxy resin (Sigma Epoxy Embedding Medium Kit). Using a Leica EMUC7 ultramicrotome, epoxy blocks were cut into ultrathin sections (70 nm thick) and contrasted with uranyl acetate and lead citrate. Ultrastructural studies were performed under an electron transmission microscope Jeol JEM 2100 (80 kV) and EM-MENU 4 (TVIPS) software, repeated on three different individuals from each species, for a total of 126 individuals.

## RESULTS

### Eurymelinae symbiotic systems

**Fig 1:**
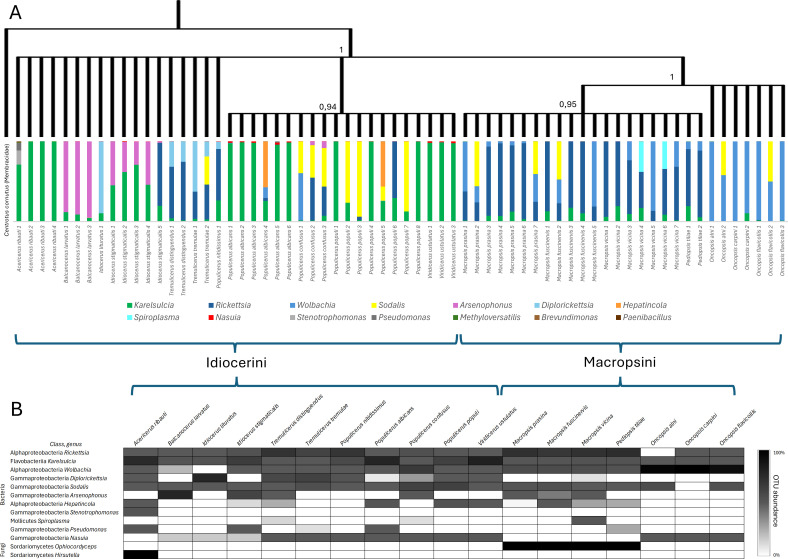
Eurymelinae symbiotic systems, 16S rRNA metabarcoding. (**A**) Cladogram based on Bayesian analysis of the most abundant sequences of the bacteria *Karelsulcia* for each individual, showing phylogeny among Eurymelinae species. Posterior probabilities are shown above the nodes. The outgroup is *Karelsulcia* from *Centrotus cornutus* (MN082139.1) (50). Below the cladogram, the graphs show the percentage of the relative abundance of bacteria for each sample based on the number of sequence reads. (**B**) Relative abundance of OTUs of both bacterial and fungal microorganisms, collectively for each Eurymelinae species.

We examined the composition of the microbiome for the following 18 Eurymelinae species: *Acericerus ribauti*, *Balcanocerus larvatus*, *Idiocerus lituratus*, *Idiocerus stigmaticalis*, *Macropsis fuscinervis*, *Macropsis prasina*, *Macropsis vicina*, *Oncopsis alni*, *Oncopsis carpini*, *Oncopsis flavicollis*, *Pediopsis tiliae*, *Populicerus albicans*, *Populicerus confusus*, *Populicerus nitidissimus*, *Populicerus populi*, *Tremulicerus distinguendus*, *Tremulicerus tremulae*, and *Viridicerus ustulatus*. The presence of *Karelsulcia* bacteria was observed in all 66 examined individuals (Fig. 1A and B). Based on the sequences of these bacteria, we created a cladogram that divides the Eurymelinae leafhoppers into three distinct groups. The first is mainly composed of representatives of the genera *Acericerus*, *Balcanocerus*, *Idiocerus,* and *Tremulicerus* (tribe Idriocerini); the second includes *Populicerus* and *Viridicerus* (tribe Idiocerini); and the third consists of *Macropsis*, *Pediopsis*, and *Oncopsis* (tribe Macropsini) (Fig. 1A and 5). Betaproteobacteria *Nasuia* were found in all tested leafhoppers, except for the representatives of the genera *Acericerus*, *Macropsis*, and *Pediopsis*. However, in the body of the mentioned genera, symbiotic fungi were present: *Hirsutella* in *Acericerus* insects and *Ophiocordyceps* in *Macropsis* and *Pediopsis* insects. Alphaproteobacteria of the genera *Wolbachia* and *Rickettsia* are present in almost all the examined leafhoppers (Fig. 1A and B). In some insects, mainly the species of the genus *Tremulicerus* and *I. lituratus*, we demonstrated the presence of *Diplorickettsia* bacteria. *Sodalis* gammaproteobacteria were relatively common microorganisms, with a large number of sequence reads obtained from some representatives of the species *T. tremulae*, *P. confusus*, *P. populi*, *M. prasina*, *M. fuscinervis*, *O. alni*, and *O. flavicollis* (Fig. 1A and B). The genus *Idiocerus* (except *I. lituratus*) and the species *P. confusus* are characterized by the presence of gammaproteobacteria from the genus *Arsenophonus*. In addition to the microorganisms mentioned above, we reported in individual cases the presence of the following bacteria: *Hepantincola*, *Spiroplasma*, *Stenotrophomonas*, and *Pseudomonas* (Fig. 1A and B).

**Fig 2:**
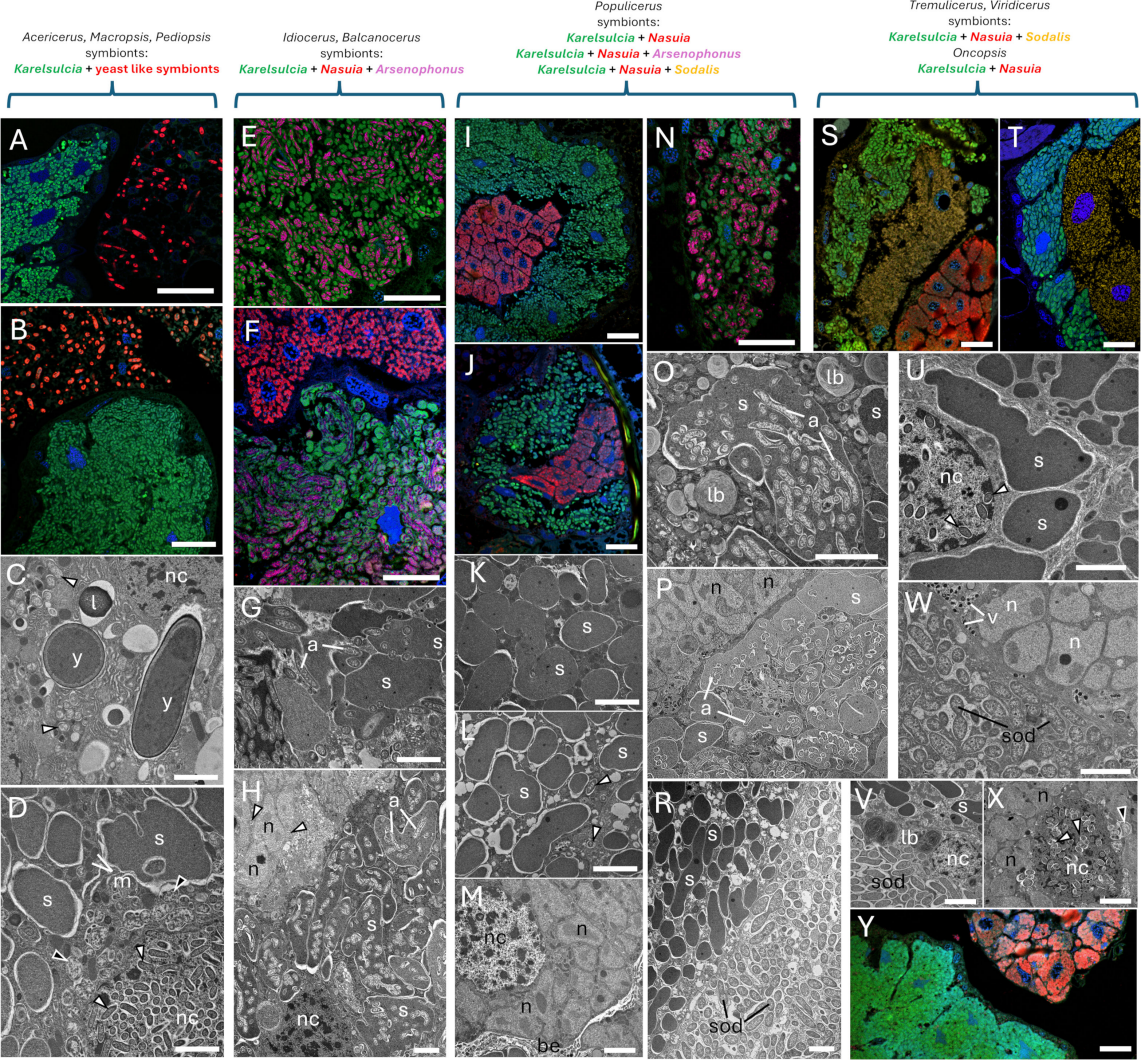
Eurymelinae symbiotic systems. (**A**) *A. ribauti*, bacteriome containing *Karelsulcia* bacteria (green), surrounded by a fat body where yeast-like symbionts of the genus *Hirsutella* are present (red). (**B**) *M. vicina*, bacteriome containing *Karelsulcia* bacteria (green), surrounded by a fat body where yeast-like symbionts of the genus *Ophiocordyceps* (red) are present. (**C**) *M. prasina*, *Ophiocordyceps* yeast-like symbionts (y) in insect fat body. (**D**) *P. tiliae, Karelsulcia* bacteria (s) in bacteriocyte; note *Wolbachia* bacteria (white arrowheads) inside the cell nucleus (nc). (**E and F**) *I. stigmaticalis*, *Arsenoponus* bacteria (purple) residing inside *Karelsulcia* bacteria (green); *Nasuia* bacteria (red) present in bacteriocytes forming a separate zone. (**G**) *B. larvatus*, *Arsenoponus* bacteria (a) infecting *Karelsulcia* bacteria (s). (**H**) *I. stigmaticalis*, bacteriocytes filled with *Karelsulcia* bacteria (s) infected by *Arsenophonus* bacteria (a); in the upper left corner, a fragment of a bacteriocyte occupied by *Nasuia* bacteria (n). (**I**) *P. albicans* and (**J**) *P. populi*, bacteriome composed of two zones—bacteriocytes filled with *Karelsulcia* bacteria (green) and *Nasuia* bacteria (red). (**K**) *P. albicans* and (**L**) *P. populi*, pleomorphic *Karelsulcia* bacteria inhabiting bacteriocytes. (**M**) *P. albicans*, the fragment of a bacteriocyte filled with pleomorphic *Nasuia* bacteria (n). (**N**) *P. confusus,* bacteriocytes filled with *Karelsulcia* bacteria (green) infected by *Arsenophonus* bacteria (purple). (**O and P**) *P. confusus, Karelsulcia* bacteria (s) filled with many rod-shaped *Arsenophonus* bacteria (a); in the upper left corner, the fragment of a bacteriocyte occupied by *Nasuia* bacteria (n). (**R**) *P. nitidissimus*, two zones of a bacteriome: pleomorphic *Karelsulcia* bacteria (s) (left) and rod-shaped *Sodalis* bacteria (sod) (right). (**S**) *T. tremulae* and (**T**) *V. ustulatus*, bacteriome where bacteriocytes filled with various bacteria form three distinct zones: *Karelsulcia* (green), *Sodalis* (yellow), and *Nasuia* (red). (**U**) *T. distinguendus,* pleomorphic *Karelsulcia* bacteria (s) inhabiting bacteriocytes; note rod-shaped alphaproteobacteria present in the cell nucleus (white arrowheads). (**W**) *T. distinguendus*, two zones of bacteriome: rod-shaped *Sodalis* bacteria (sod) (left) and pleomorphic *Nasuia* bacteria (n) (right); note the viruses (v) residing in the bacteriome epithelium between zones. (**V**) *V. ustulatus,* two zones of bacteriome: pleomorphic *Karelsulcia* bacteria (s) (left) and rod-shaped *Sodalis* bacteria (sod) (right). (**X**) *O. alni*, pleomorphic *Nasuia* bacteria (n) inhabiting bacteriocytes; note rod-shaped alphaproteobacterial present in the cell nucleus (white arrowheads). (**Y**) *O. flavicollis*, bacteriocytes filled with *Karelsulcia* (green) and *Nasuia* (red) bacteria forming two separate bacteriomes. (**A, B, E, F, I, J, N, S, T, Y**) Confocal microscope; cell nuclei, DAPI staining (blue); scale bar: 40 µm. (**C, D, G, H, K–M, O–R, U–X**) TEM; be, bacteriome epithelium; black arrowheads, rod-shaped gammaproteobacterium; l, lipid droplet; lb, lamellar body; m, mitochondrion; nc, cell nucleus; white arrowhead, rod-shaped alphaproteobacterium; scale bar: 3 µm.

During microscopic analysis, we observed the presence of specialized organs called bacteriomes, composed of mostly spherical cells called bacteriocytes, which serve as the habitat for obligate symbionts (Fig. 2A, B, E, F, I, J, N, S, T and Y; Movies S1 to S3). Bacteriomes appeared as two pairs of structures located on either side of the body, in the upper part of the abdomen near the reproductive organs. They were surrounded by a single-layered epithelium called the bacteriome sheath. In the bodies of the representatives of the genera *Acericerus*, *Macropsis*, and *Pediopsis*, we noted the occurrence of bacteriomes occupied only by the bacteria *Karelsulcia*. Additionally, yeast-like microorganisms were present in the insect’s fat body (Fig. 2A to D; Movie S1). In other leafhoppers, the bacteriomes formed zones, each surrounded by its own epithelium and composed of bacteriocytes whose cytoplasm was filled with specific types of bacteria: *Karelsulcia* and *Nasuia*. *Karelsulcia* bacteriocytes formed the outer zone of bacteria, while *Nasuia* bacteriocytes filled the central part (Fig. 2E, F, I and J; Movie S2). In the members of the genus *Oncopsis*, bacteriocytes of *Karelsulcia* and *Nasuia* formed separate bacteriomes (Fig. 2Y). Bacteria of the genus *Karelsulcia* were quite large microorganisms (10–12 µm in length), pleomorphic, sometimes slightly elongated, electron-dense, appearing darker in electron microscopy images in contrast to *Nasuia* bacteria (Fig. 2D, G, K, L, R and U). Betaproteobacteria *Nasuia* also had a pleomorphic shape, but unlike *Karelsulcia* bacteria, they were more rounded (Fig. 2H, W and X), sometimes branching (Fig. 2M and P), and were electron-transparent in TEM images.

In individuals of the species *B. larvatus*, *I. stigmaticalis*, and *P. confusus*, we observed an unusual symbiotic system. The gammaproteobacteria *Arsenophonus* were present inside the bacteria of the genus *Karelsulcia* (Fig. 2E to H and N to P; Movie S3). Rod-shaped, strongly elongated *Arsenophonus* bacteria did not settle on all *Karelsulcia* bacteria. Some of them occurred in the cytoplasm of *Karelsulcia* bacteriocytes (Fig. 2G, H, O and P). These free bacteria were often removed by lysosomes. This is why we observed, in some places, the formation of lamellar bodies as a result of the destruction of bacteria recognized as pathogens (Fig. 2O). In turn, rod-shaped gammaproteobacteria *Sodalis* formed a separate zone between *Karelsulcia* and *Nasuia* within the bacteriomes of their hosts: *P. nitidissimus*, *T. tremulae*, *T. distinguendus*, and *V. ustulatus* (Fig. 2R, S, T, W and V). We sometimes observed *Sodalis* bacteria and viruses in the epithelium separating the individual zones, with lamellar bodies visible in the places where they were destroyed (Fig. 2W and V).

**Fig 3:**
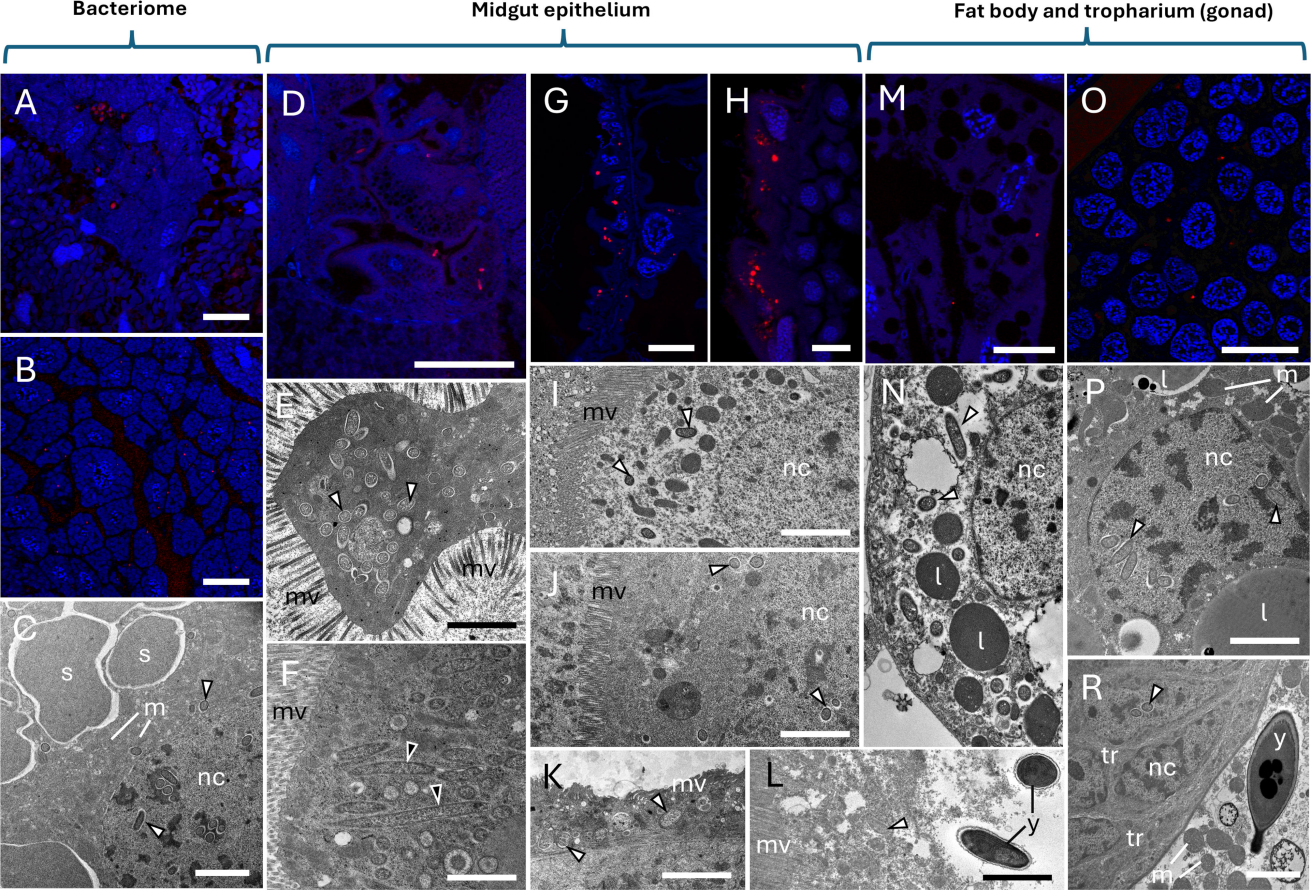
Facultative bacteria in various tissues of Typhlocybinae and Eurymelinae insects. (**A**) *P. populi *and (**B**) *P. albicans*, *Wolbachia* bacteria (red) present in *Nasuia* bacteriocytes zone. (**C**) *V. ustulatus*, alphaproteobacteria (white arrowheads) occurring in the cytoplasm and nucleus (nc) of *Karelsulcia* (s) bacteriocyte. (**D**) *E. aurata*, *Wolbachia* bacteria (red) present in the midgut epithelium. (**E**) *F. citrinella*, alphaproteobacteria (white arrowheads) settling the cell of the midgut epithelium. (**F**) *E. lethierryi*, gammaproteobacteria (black arrowheads) present in the midgut epithelium. (**G**) *P. albicans* and (**H**) *V. ustulatus, Wolbachia* bacteria (red) occurring in the midgut epithelium. (**I**) *Z. hyperici* (**J**), *V. ustulatus* (**K**), *T. quercus* (**L**), and *M. vicina*, alphaproteobacteria (white arrowheads) present in the midgut epithelium. (**M**) *E. calcarata*, *Wolbachia* bacteria (red) in insect fat body. (**N**) *F. citrinella*, alphaproteobacteria (white arrowheads) settling in fat body cells. (**O**) *P. albicans*, *Wolbachia* bacteria (red) occurring in trophocytes (ovary). (**P**) *T. tremulae*, alphaproteobacteria (white arrowheads) settling in the nucleus of fat body cells. (**R**) *P. tiliae,* alphaproteobacteria (white arrowheads) in trophocytes (tr) (ovary); note yeast-like microorganism (y) in the fat body, lower right corner. (**A, B, D, G, H, M, O**) Confocal microscope; cell nuclei, DAPI staining (blue); scale bar: 40 µm. (**C, E, F, I-L, N, P, R**) TEM; l, lipid droplet; m, mitochondrion; mv, intestinal microvilli; nc, cell nucleus; y, yeast-like microorganism; scale bar: 3 µm.

Representatives of alphaproteobacteria, such as *Wolbachia* and *Rickettsia*, occurred in many locations in the bodies of the studied insects. These bacteria were present inside the bacteriomes (Fig. 2D, H, L, U, X and 3A to C), but also in the fat body (Fig. 2C and 3P), midgut epithelium (Fig. 3G, J and L), and reproductive organs—specifically, the tropharia in the ovaries of females (Fig. 3O and R). Bacteria of the genera *Wolbachia* and *Rickettsia* were indistinguishable in TEM images, due to their very similar morphology: small, rod-shaped, slightly elongated, and electron-transparent. It is worth mentioning that alphaproteobacteria were quite often found inside the cell nuclei of various cell types: bacteriocytes containing both *Karelsulcia* and *Nasuia* (Fig. 2D, U, X and 3C), midgut epithelium (Fig. 3J), fat body (Fig. 3P), and trophocytes (Fig. 3R). We assumed that the above-mentioned bacteria were *Wolbachia* because this is the genus that has been previously described inside cell nuclei (30). Additionally, apart from alphaproteobacteria, we observed yeast-like fungal symbionts in the cytoplasm of the midgut epithelium of *M. vicina* insects (Fig. 3L).

### Typhlocybinae symbiotic systems

**Fig 4:**
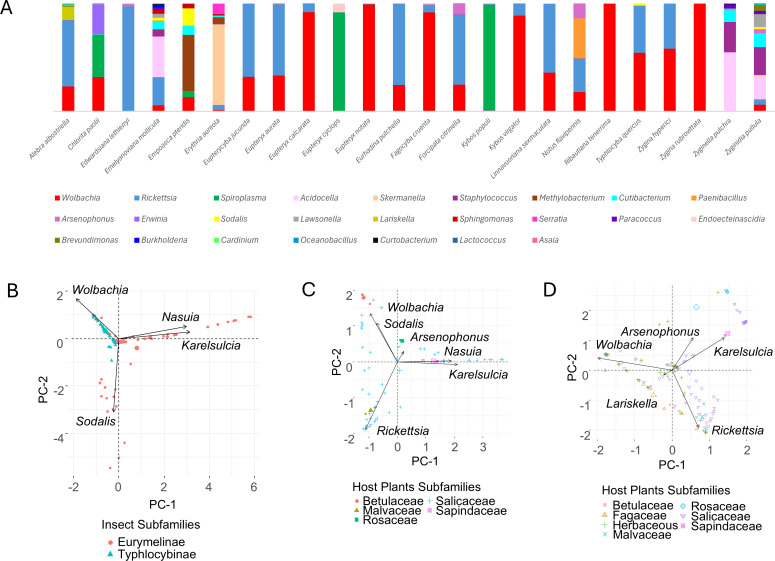
Typhlocybinae microorganisms composition and its comparison to Eurymelinae microbial system. (**A**) The percentage relative abundance of bacteria for Typhlocybinae species based on the number of sequence reads. (**B**) The PCA analysis of Typhlocybinae and Eurymelinae microbiome diversity; the arrows indicate the loadings—the most important types of bacteria differentiating two groups of insects. (**C**) The PCA analysis of Eurymelinae microbiome diversity for various plant subfamilies of which the insects feed; the arrows indicate the loadings—the most important types of bacteria differentiating host plant groups. (**D**) The PCA analysis of Typhlocybinae and Eurymelinae microbiome diversity for various plant subfamilies of which the insects feed; the arrows indicate the loadings—the most important types of bacteria differentiating host plant groups.

We examined the composition of the microbiome for the following 24 Typhlocybinae species: *Alebra albostriella*, *Chlorita paolii*, *Edwardsiana lethierryi*, *Emelyanoviana mollicula*, *Empoasca pteridis*, *Erythria aureola*, *Eupterycyba jucunda*, *Eupteryx aurata*, *Eupteryx calcarata*, *Eupteryx cyclops*, *Eupteryx notata*, *Eurhadina pulchella*, *Fagocyba cruenta*, *Forcipata citrinella*, *Kybos populi*, *Kybos virgator*, *Linnavuoriana sexmaculata*, *Notus flavipennis*, *Ribautiana tenerrima*, *Typhlocyba quercus*, *Zygina hyperici*, *Zygina rubrovittata*, *Zyginella pulchra*, and *Zyginidia pullula. Wolbachia* bacteria were present in almost all species, except *E. lethierryi, E. cyclops,* and *Z. pulchra*. The second most common bacterium was *Rickettsia* (Fig. 4A; Fig. S1A). Alphaproteobacteria were observed mainly in the insect midgut epithelium (Fig. 3D to F and K) and fat body (Fig. 3M and N). *E. cyclops* and *K. populi* were infected with *Spiroplasma* bacteria. We detected bacteria of the genus *Acidocella* in higher amounts in individuals of the species *E. mollicula*, *Z. pulchra*, and *Z. pullula*. We did not detect the presence of obligate symbionts in the samples. However, we have noted the occurrence of some facultative symbionts*: Arsenophonus*, *Sodalis*, *Lariskella*, *Serratia*, *Cardinium*, and *Asaia* (Fig. 4A). The samples also included bacteria from chitinous cuticles or the guts of insects.

To demonstrate which bacteria have the greatest impact on the differences in the microbiome of the two studied groups, based on their abundance, principal component analysis (PCA) was performed (Fig. 4B). The bacteria that have most influenced the variability of the microbiome of these groups are *Wolbachia*, *Karelsulcia*, *Nasui*a, and *Sodalis*. Similar analyses were performed to show which bacteria have the greatest impact on the differences in the microbiome of insects occurring on different host plants. One analysis was performed for Eurymelinae alone. The bacteria that most influenced the variability were *Wolbachia*, *Sodalis*, *Arsenophonus*, *Nasuia*, Karelsulcia, and *Rickettsia* (Fig. 4C). Another analysis was performed for representatives of both subfamilies simultaneously. In this case, the most influential bacteria were Karelsulcia, *Arsenophonus*, *Wolbachia*, *Lariskella*, and *Rickettsia* (Fig. 4D).

Almost all statistical models performed indicated a statistically significant role of the insect diet as a driver of microbial abundance (Table S3). Diet explained 15% to 33% of the variation in microbiome abundance (depending on the model used). Comparison using the Akaike Information Criterion (AIC), which is an estimator of the relative quality of statistical models, indicates that the most reliable PERMANOVA models take into account the more precise affiliation of the plant (to a genus, not a group corresponding to a family in most cases), explaining 33% of variance in microbiome composition, whereas the most reliable GLMM takes into account more precise (nested) block design for random effect (insect subfamily:taxa). A very low *P*-value associated with Chisq in ANOVA tests for GLMMs indicates that the models are highly useful in explaining microbial abundance. The results of dbRDA performed for both diet plant and insect taxonomical identity as explanatory variables indicate the stronger role of diet plant (33%) than insect genus (21%) or insect subfamily (3%) affiliation. However, when we control for the effects of taxonomic affiliation of insect (subfamily), the model explains only 15–28% of the overall variance in microbiome abundance.

### Phylogenetic results

**Fig 5:**
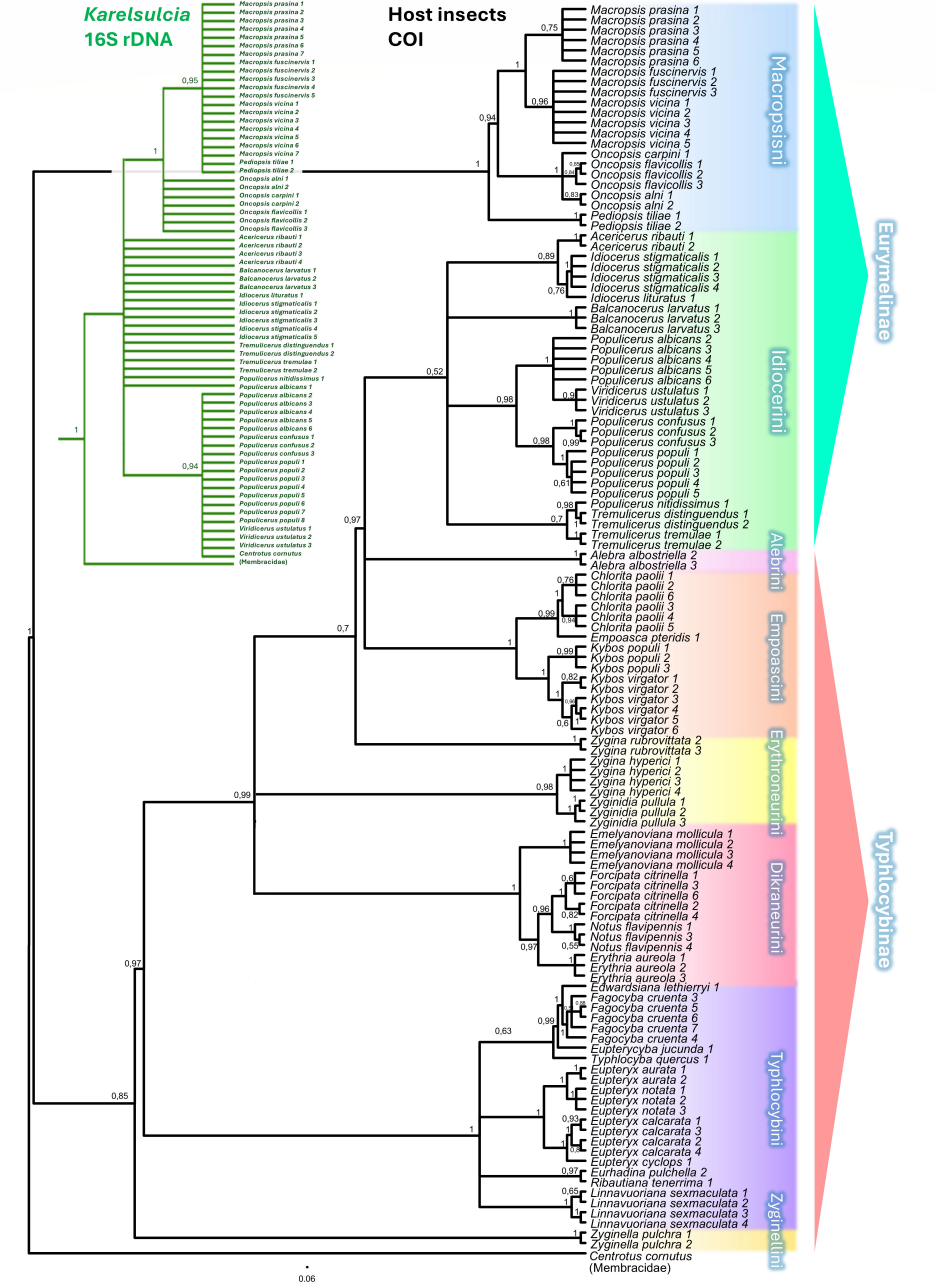
Cladograms based on Bayesian analysis of bacteria *Karelsulcia* 16S rDNA and COI insect gene, showing cophylogeny between Eurymelinae and their *Karelsulcia* symbiont (left side) and phylogeny between both Typhlocybinae and Eurymelinae species (right side). Posterior probabilities are present above the nodes. Outgroup *Karelsulcia* of *Centrotus cornutus* (MN082139.1) (50); for COI – *C. cornutus* (MW536003.1) (51).

The phylogeny of Eurymelinae, based on sequences of the bacteria *Karelsulcia*, is shown in Fig. 1A and described at the beginning of this chapter. Unfortunately, the lack of obligate symbionts in individuals of Typhlocybinae has prevented this procedure from being performed in this group. However, based on the sequence of the insect COI (cytochrome C oxidase I) gene, we have constructed a cladogram showing the relationships between all studied leafhoppers (Fig. 5). Representatives of the Macropsini tribe constitute a separate group from other leafhoppers. The remaining members of Eurymelinae form a separate clade within Typhlocybinae leafhoppers, showing closest relationships to members of the genera *Alebra*, *Chlorita*, *Empoasca*, and *Kybos*. The remaining genera of Typhlocybinae form successive clades among themselves within tribes and genera. The most outlying group is formed by representatives of the species *Z. pulchra*.

### Transovarial transmission of microorganisms

**Fig 6:**
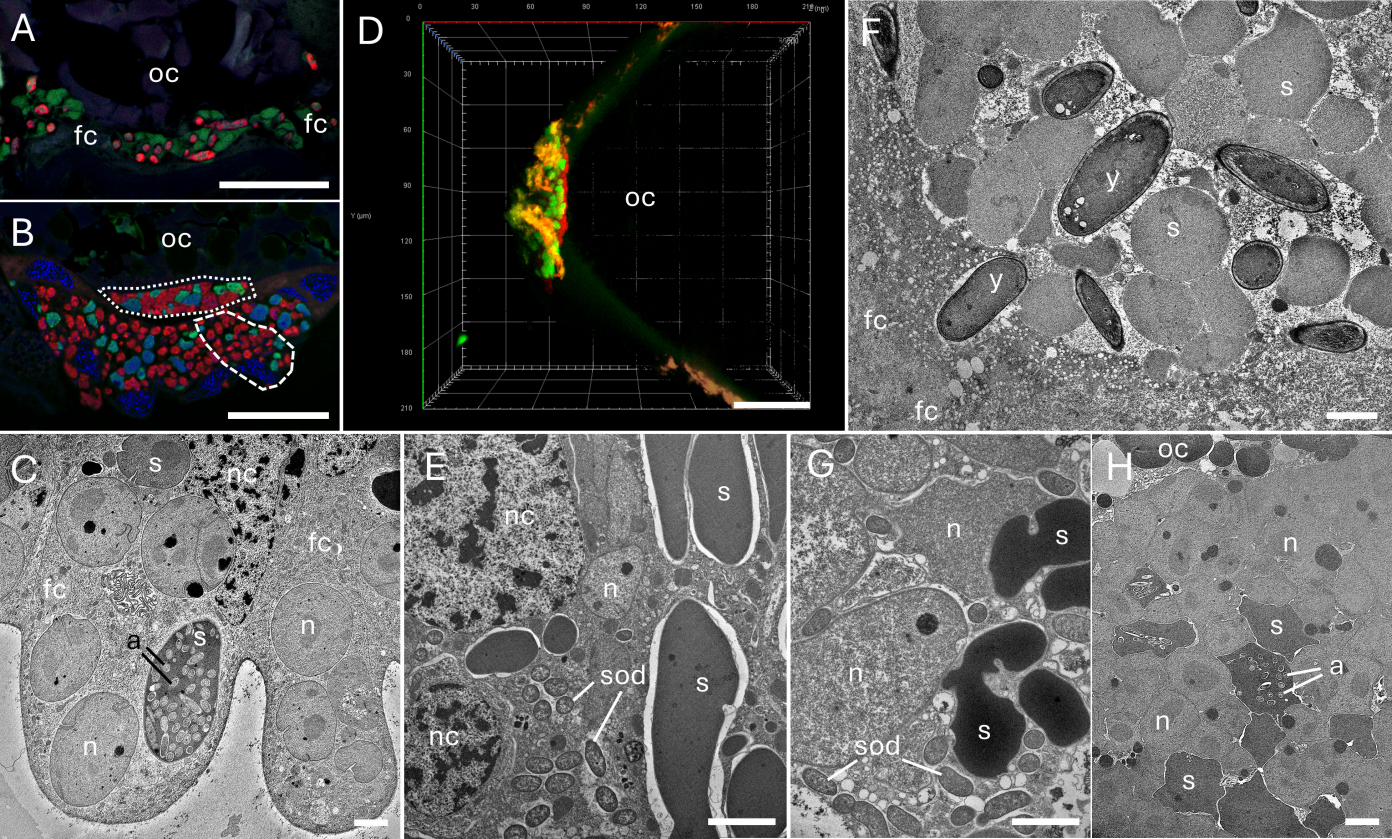
Transovarial transmission of bacteria in Eurymelinae leafhoppers. (**A**) *M. vicina*, *Karelsulcia* bacteria (green) and *Ophiocrodyceps* yeast-like symbionts (red) infecting follicular cells (fc) surrounding the posterior pole of the oocyte (oc). (**B**) *P. albicans*, *Karelsulcia* (green) and *Nasuia* (red) bacteria infecting follicular cells (one of them marked by a dashed line) and forming cap-like “symbiont ball” (dotted line) in an invagination of oolemma. (**C**) *B. larvatus*, *the* fragment of follicular cells (fc) filled with *Karelsulcia* (s) and *Nasuia* (n) bacteria; note *Arsenophonus* bacteria (a) inside *Karelsulcia* (s). (**D**) *P. albicans,* 3D view of a “symbiont ball” consisted of *Karelsulcia* (green) and *Nasuia* (red); note the faint green and red flashes of autofluorescence on the chorion. (**E**) *T. tremulae*, the three types of bacteria: *Karelsulcia* (s), *Sodalis* (sod), and *Nasuia* (n) present in follicular cells. (**F**) *M. prasina*, the fragment of a “symbiont ball” consisted of *Karelsulcia* bacteria (s) and *Ophiocordyceps* yeast-like symbionts (y); note follicular cell (fc) in the bottom left corner. (**G**) *V. ustulatus*, *the* fragment of a “symbiont ball” consisted of *Karelsulcia* (s), *Sodalis* (sod), and *Nasuia* (n) bacteria. (**H**) *B. larvatus*, the fragment of a “symbiont ball” consisted of *Karelsulcia* (s) and *Nasuia* (n) bacteria; note *Arsenophonus* bacteria (a) inside *Karelsulcia* (s); note the fragment of an oocyte (oc) in the upper left corner. (**A, B, D**) Confocal microscope; cell nuclei, DAPI staining (blue); oc, oocyte; scale bar: 40 µm. (**C, E, F through H**) TEM, nc, follicular cell nucleus; scale bar: 3 µm.

During microscopic observations, we observed transovarial transmission of symbiotic microorganisms only in representatives of the Eurymelinae subfamily. Symbiotic bacteria migrate from the bacteriome towards the posterior pole of the terminal oocyte, located at the end of the ovariole—the basic component of the ovary. If we observed symbiotic fungi in species, we noted their migration from the fat body to the oocytes (Fig. 6A). The symbionts infect follicular cells, filling their cytoplasm (Fig. 6A to C and E). During this process, *Karelsulcia* and *Nasuia* bacteria change their shape from pleomorphic (still in Fig. 6E) to more spherical (Fig. 6B, C and F). We have not noted this phenomenon for symbiotic fungi (Fig. 6F). Then, the microorganisms enter the space between the oocyte and the follicular cells, called the perivitelline space. At this point, symbiotic microorganisms form a structure called a “symbiont ball,” which in this case has a “cap-like” shape (Fig. 6B and D; Movies S4 and S5). Within the “symbiont ball,” we observed symbionts that were present in the host bacteriome, e.g., *Karelsulcia*, *Nasuia*, *Sodalis* in *V. ustulatus* (Fig. 6G), or *Karelsulcia*, *Arsenophonus*, and *Nasuia* in *B. larvatus*. *Arsenophonus* bacteria were also present inside the *Karelsulcia* bacteria within the bacteriome (Fig. 6H).

## DISCUSSION

### Microbiome comparison

Despite their phylogenetic relationship, the leafhoppers from the subfamilies Typhlocybinae and Eurymelinae differ in their microbiome (Fig. 1A and 4A). We noticed that most of the Eurymelinae individuals showed higher microbial diversity compared to Typhlocybinae (Fig. S1B and C—alpha diversity, Chao and Shannon index). Considering obligate symbionts as those present in all individuals of a given species, transmitted transovarially, we confirm that Typhlocybinae is a group of leafhoppers devoid of obligate symbiotic microorganisms and with poorer microbiome diversity. The studied Eurymelinae insects are monophagous, living in trees and feeding on phloem sap, while the Typhlocybinae suck up the mesophyll contents, having their host plants and trophic specialty more diverse (Table S1). That is probably why the variability of their microorganisms largely comes down to the diversity of facultative bacteria and gut microflora. Nevertheless, some of the examined species of both subfamilies feed on the same host plant, e.g. host-plant *Carpinus betulus* inhabiting leafhoppers *O. carpini* versus *F. cruenta*; host*-*plant *Salix alba* inhabiting leafhoppers *I. stigmaticalis* versus *E. pteridis, K. virgator*; host-plant *Salix cinerea* inhabiting leafhoppers *M. prasina, P. confusus* versus *L. sexmaculata*. However, in these cases, we noticed only common facultative alphaproteobacteria, *Wolbachia* or *Rickettsia*, which are presumably transmitted horizontally (Fig. 1A and 4A).

Phylogenetic position along with diet may influence the host microbiome (52). The occurrence of some bacteria in the insects Typhlocybinae and Eurymelinae was dependent on the host plant. In the Eurymelinae group, the main bacteria influencing the grouping of leafhoppers according to their host plants are *Karelsulcia*, *Nasuia*, *Arsenophonus*, *Sodalis*, *Rickettsia*, and *Wolbachia* (Fig. 4C). When analyzing two subfamilies together, we observed such trends as the presence of *Rickettsia* bacteria was higher in the insects living on Salicaceae family trees, *Wolbachia* in leafhoppers on herbaceous plants, *Arsenophonus* in insects on Rosaceae plants, and *Lariskella* in leafhoppers on Fagaceae plants (Fig. 4D). We implemented a series of different statistical tests to determine if microbial community abundance was significantly associated with the most common diet plants. All these results indicate that although diet does not fully explain the variability, it is still an important factor, alongside the taxonomical affiliation of insects (host), influencing microbiome abundance (Table S3).

We should point out that the Eurymelinae microbiome is mainly composed of co-symbionts, probably responsible for the synthesis of essential amino acids. This is a similar situation to other members of the Cicadellidae family, mainly the subfamily Deltocephalinae (19, 30, 50, 53–63). The main co-symbionts of Eurymelinae are *Karelsulcia* and betaproteobacteria *Nasuia*. They are also the main bacteria that, together with *Sodalis* and *Wolbachia*, distinguish the Eurymelinae microbiome from Typhlocybinae (Fig. 4B). The tribe Macropsini is a distinctly different group—the content of *Karelsulcia* bacteria is lower (Fig. 1A), the bacteriomes are smaller (Movie S2), the symbiotic system is supplemented with *Ophiocordyceps* yeast-like microorganisms and occasionally *Sodalis* bacteria, and individuals from the genus *Oncopsis* do indeed have betaproteobacteria *Nasuia*, but they inhabit separate bacteriomes (Fig. 2Y). The presence of fungi as symbiotic partners of leafhoppers has been observed previously both in Cicadomorpha and Fulgoromorpha (57, 64, 65). In this group, we noted that alphaproteobacteria often appeared in large quantities. *Wolbachia* was more common in individuals from the *Macropsis* genus, while *Rickettsia* predominated in those from the genus *Oncopsis* (Fig. 1A). It should be added that these insects differ in their diet. *Oncopsis* insects feed on the sap of trees from the Betulaceae subfamily, while *Macropsis* feed on Salicaceae. The remaining representatives classified as the tribe Idiocerini can be divided into two groups due to their microbiomes. The first, more uniform, phylogenetically coherent group consists of the genera *Populicerus* and *Viridicerus* (middle clade Fig. 1A). These leafhoppers have a symbiotic system consisting of *Karelsulcia + Nasuia*, supplemented in some cases by *Sodalis* bacteria. The exceptions here are individuals of the species *P. confusus*—their systems have numerous alphaproteobacteria and *Arsenophonus* bacteria inside *Karelsulcia* associates. These insects live on plants of the *Salix cinerea* species, unlike the others from this group, which feed on trees of the genus *Populus*. The *Arsenophonus* bacterium is considered to be a symbiont that, under stressful conditions or with a smaller amount of food, displaces obligate symbionts, replacing them in the synthesis of amino acids (66). It may also be responsible for the synthesis of vitamin B (67). The occurrence of bacteria in bacteria has already been described (58, 60, 68). The last group of Eurymelinae, distinguished by us due to the microbiome, contains diverse systems in which, in addition to *Karelsulcia* bacteria, the following can be found: (i) symbiotic *Hirsutella* fungi in individuals of the genus *Acericerus* (the only insects among those studied feeding on plants of the *Acer* genus); (ii) *Arsenophonus* bacteria inside *Karelsulcia* bacteria in individuals of the genera *B. larvatus* and *I. stigmaticalis*; and (iii) *Diplorickettsia* bacteria in representatives of the genus *Tremulicerus* and species *I. lituratus* (Fig. 1A and B). Some *Diplorickettsia* bacteria have been described as human pathogens—microorganisms occurring in the genus *Ixodes* (69, 70). *Tremulicerus* insects are also characterized by having significant amounts of *Rickettsia* bacteria in their bodies. We noticed that two *Macropsis* insects were infected with *Spiroplasma* bacteria (greater number of reads). *Spiroplasma* is a microorganism that can be considered as a pathogen, commensal bacteria, or even mutualistic partner. Their influence has been compared to the *Wolbachia* bacteria (71, 72). Also, two individuals, but from the genus *Populicerus*, were infected with the bacterium *Hepanticola*, an extracellular midgut bacterium found in terrestrial isopods that feeds on the nutrients of its host (73). In some cases, the sequencing results obtained by us revealed bacteria, likely collected from the surface of the insect cuticle or occurring in their digestive tract, e.g., *Stenotrophomonas*, *Pseudomonas*, and *Brevundimonas*. We do not rule out that the individual infections described above may originate from parasites or parasitoids inhabiting the insect’s body, although our samples were checked for such organisms during preparation, including COI gene sequencing.

In contrast to Eurymelinae, the body of insects from the subfamily Typhlocybinae is inhabited mainly by alphaproteobacterial microorganisms of the genera *Wolbachia* and *Rickettsia. Wolbachia* and *Rickettsia* are bacteria often classified as intracellular commensals or pathogens. They inhabit various types of host cells, quite frequently occupying the host bacteriomes or gonads, which may provide greater shelter by isolating them from the rest of the body and enabling potential vertical transmission. It persists in the population and is passed on to the greatest extent by females to offspring. *Wolbachia* bacteria are known to interfere with sex determination in their hosts, causing feminization, male offspring death, and induction of parthenogenesis (74). *Wolbachia* might also have a positive effect on its host, such as counteracting viruses and participating in the production of vitamin B for blood-sucking insects (75–79). *Rickettsia* bacteria are most often transmitted horizontally by parasites and also inhabit places such as the ovaries, mouthparts, and digestive tract. This microorganism has a positive effect on fertility, survival rate, or accelerated development, but also on the feminization of offspring (80).

In some Typhlocybinae species, we observed the presence of bacteria that can be considered as facultative symbionts, such as *Spiroplasma*, *Acidocella*, *Arsenophonus*, *Sodalis*, *Lariskella*, *Serratia*, *Cardinium*, and *Asaia*. Representatives of the species *Ch. paolii*, *E. pteridis*, and especially *E. cyclops* and *K. virgator* possess *Spiroplasma* bacteria in their bodies. We obtained a relatively large number of *Acidocella* reads from samples originating from *E. mollicula*, *Z. pulchra,* and *Z. pullula*. These bacteria have been described in the guts of bees (81). Moreover, they belong to the Acetobacteriaceae, a group of bacteria among which bacterial symbionts, such as *Asaia,* can be found, which are distributed in the guts, fatty bodies, and salivary glands, and are involved in the host’s nutrient supply (82). Also, *Arsenophonus* bacteria were detected in some samples, with higher read numbers in *F. citrinella* and *N. flavipennis* individuals from the same habitat, *Carex* plants, suggesting a possibility of horizontal transport. We have detected *Sodalis* bacteria among *E. mollicula, E. pteridis,* and *T. quercus*. These microorganisms, as we know, are described not only as facultative but also as obligate symbionts involved in the nutrition of the host, associated with the bacteriome (83). However, we did not observe any bacteriomes during a histological examination of Typhlocybinae. All tested individuals of the species *A. albostriella* harbor bacteria of the genus *Lariskella* in their bodies. It is an *Ixodes* symbiont, the quantity of which varies mainly depending on the sex of the individuals (84). *Serratia* bacteria occurred in the species *E. aureola,* and *Cardinium* endosymbiont in *Z. pullula*, both of which were described as facultative symbionts of Cicadomorpha (50, 56). The remaining detected microorganisms, often observed in single samples, were extracellular bacteria, probably collected from the insect’s cuticle surface or present in the host guts.

### Causes of symbiont loss in Typhlocybinae

The differences in the symbiotic systems of the two compared leafhopper groups can have many causes. Although they inhabit similar habitats, and sometimes even the same plants, they differ in the composition of microorganisms living in their bodies. One of the potential causes could be the fact that the insects obtain nutrients from different plant tissues, which differ in their composition—specifically, the content of essential amino acids. However, the question remains why the ancestors of Typhlocybinae shifted their diet and ended cohabitation with their co-symbionts. This could have been caused by competition among other leafhoppers for the same host plants and for the same ecological niche. Another scenario may be related to their body size. Typhlocybinae insects, sometimes called microleafhoppers, due to their smaller body size, may have had difficulty competing with other bigger leafhoppers and also with puncturing into deeper parts of plant tissues. Other possible scenarios of obligate symbionts loss are the reduction of their genome. On the other hand, a sudden loss of nutritional symbionts would lead to the death of the insect, so we assume that these changes occurred simultaneously with the diet shift. There is also a possibility that changes in the composition of symbionts might influence the adaptation of the host to certain external factors, such as temperature (42, 85). Obligate symbionts with reduced genomes could prevent adaptation to changes in environmental conditions (40, 42, 43). This hypothesis fits in well with the fact that representatives of Typhlocybinae more often inhabit tropical zones. A gradual change from phloem sap to mesophyll combined with a change in the symbiotic system in Typhlocybinae insects could have influenced the fact that they show greater host plant variability and trophic specialization. Within this group, both mono-, oligo-, and polyphagous insects are found (Table S1). In order to understand the influence of the lack of obligate symbionts and the possession of facultative ones on adaptation to different environmental conditions, tests on insects, e.g., fitness assays, should be performed. In the case of facultative microorganisms, they could be combined with symbiont manipulation. Populations infected with specific genera of bacteria should be created.

The traditional division of symbionts into obligate and facultative may not be entirely correct, because there are bacteria that are difficult to recognize as one or the other. These include *Sodalis* and *Arsenophonus*. On the one hand, in the case of some Eurymelinae, they are present in the bacteriomes, are transmitted transovarially, and are probably involved in the synthesis of amino acids. On the other hand, they do not occur in all individuals of a given species. They can also inhabit only the fat body. These bacteria were also detected in some Typhlocybinae insects. We could define them as facultative symbionts, but in some cases, they take on the role of an obligate symbiont. In order to better understand the role of symbionts, additional studies should be performed to examine their genomes.

### Phylogenetic remarks

Our work presents a summary of the phylogeny of the studied leafhoppers, regarding many species that were not included in previous phylogenetic analyses. The phylogeny of Eurymelinae reconstructed based on the *Karelsulcia* symbiont sequences mirrors the phylogeny based on the insect gene sequences (Fig. 1A and 5). Based on the obtained cladograms, we can state that the tribe Macropsini is a monophyletic group that separates from the other studied leafhoppers (Fig. 5). Our studies are consistent with the distinctiveness of this group, as shown in earlier works (2, 11). The remaining representatives of Eurymelinae constitute a compact clade more related to the studied Typhlocybinae. This phylogeny of Macropsini and Idiocerini coincides with that shown in the supplementary phylogenetic tree in the work of Dietrich and coworkers (2), where Macropsini is a distinct group related to the Deltocephalinae. In the past, these leafhoppers were considered a separate subfamily. Insects from the tribe Idiocerini are probably a paraphyletic group as indicated in the work mentioned above (2) and confirmed by our studies. Additionally, the diversity of this group can be seen in their symbiotic communities. In our cladogram, the insects of the tribe Idiocerini are most closely related to the genera *Alebra* (tribe Alebrini) and the genera belonging to the tribe Empoascini (*Chlorita*, *Empoasca*, and *Kybos*) (Fig. 5). Alebrini is considered to be phylogenetically the oldest group of Typhlocybinae, from which the remaining representatives of this group may have originated (86). The sister groups to all of the above-mentioned leafhoppers are Erythroneurini (genera *Zygina* and *Zyginidia*) and Dikraneurini tribe (genera *Emelyanoviana*, *Forcipata*, *Notus*, and *Erythria*). The lower part of the cladogram is filled by a large group of representatives of the Typhlocybini tribe and a small group consistent with the genus *Zyginella* (tribe Zyginellini). To summarize, the cladogram created in our study confirms the phylogenetic classification of insects within Typhlocybinae (87).

### Microorganism transmission

The transmission of microorganisms can occur horizontally or vertically, between generations. Horizontal transmission usually concerns facultative bacteria, mainly representatives of alphaproteobacteria, e.g., *Wolbachia* or *Rickettsia*. It probably occurs between populations or species of the studied Eurymelinae and Typhlocybinae insects, especially when they occur in the same location and on the same host plant. More advanced vertical transmission is performed through infections of the ovarioles (components of the female reproductive system), which mainly affects obligate symbionts. Using microscopic methods, we did not observe any traces of transovarial transport in representatives of Typhlocybinae. However, we have observed this kind of transmission in representatives of Eurymelinae, and it occurs according to a mechanism similar to that of other representatives of leafhoppers or even Auchenorrhyncha (30, 50, 60, 65). Microorganisms pass through the follicular epithelium into the perivitelline space, where they form a cap-like structure called the “symbiont ball.” This phenomenon confirms the long-term coevolution of these insects with their symbionts.

### Conclusion

In this work, based on comprehensive molecular and microscopic studies, we compare symbiotic systems of two closely related Cicadellidae subfamilies: Typhlocybinae and Eurymelinae. We confirm the hypothesis of the lack of obligate symbionts in the leafhoppers from the subfamily Typhlocybinae. As obligates, we consider the nutritional microorganisms present in bacteriomes or fungal symbionts. Since representatives of the tribe Idiocerini (Eurymelinae) are closely related to the ancestral Typhlocybinae tribe Alebrini, it is possible that the two examined subfamilies have a common ancestor. While Eurymelinae is a subfamily characterized by a large diversity of symbiotic systems, including *Karelsulcia*, *Nasuia*, *Arsenophonus*, *Sodalis* bacteria, and yeast-like fungal symbionts, Typhlocybinae members have a poor microbiome, characterized mainly by the presence of alphaproteobacteria, e.g., *Wolbachia*, which are common in the world of insects and even arthropods (88). It remains enigmatic what caused the interruption of the close mutual symbiosis and long coevolution that connected these leafhoppers with symbiotic associates. This could have been caused by many factors, starting with diet shift from phloem to mesophyll, competition between leafhoppers for host plants or ecological niches, and the reduction of the genomes of obligate symbionts. If the loss of bacteriome symbionts had a beneficial effect on temperature adaptation, species belonging to Typhlocybinae might show greater resistance to climate change (40, 42, 43). Unlike the monophagous Eurymelinae, the Typhlocybinae group includes insects with different trophic specialties, feeding on various trees, shrubs, or herbaceous plants. It is possible that the lack of dietary specialization (and therefore a wide dietary niche) also results from the lack of obligate symbionts in Typhlocybinae. We can also notice a particular relationship between the microbiome composition and the main host plant, with a large share of *Rickettsia* in leafhoppers feeding on trees belonging to the Salicaceae family. Typhlocybinae is a reasonably large group found in many habitats on our planet, and it is especially abundant in a tropical zone. This is why we intend to undertake future research on the understanding of the symbiotic systems of this group by collecting individuals from many areas and comparing the results with the data referring to the ecological niches they inhabit.

## Data Availability

Sequence data have been deposited in Figshare under the following project link: https://figshare.com/projects/Typhlocybinae_and_Eurymelinae_microbiomes/220621. Kobiałka, Michał (2024). Eurymelinae Bacteria 16S rDNA. figshare. Data set. https://doi.org/10.6084/m9.figshare.27010873.v1 Kobiałka, Michał (2024). Typhlocybinae Bacteria 16S rDNA. figshare. Data set. https://doi.org/10.6084/m9.figshare.27010912.v1 Kobiałka, Michał (2024). Eurymelinae Fungi ITS. figshare. Data set. https://doi.org/10.6084/m9.figshare.27011410.v1 Kobiałka, Michał (2024). Typhlocybinae Fungi ITS. figshare. Data set. https://doi.org/10.6084/m9.figshare.27011431.v1 Kobiałka, Michał (2024). Phylogenetic cladograms. figshare. Data set. https://doi.org/10.6084/m9.figshare.27011437.v1

## SUPPLEMENTAL MATERIAL

Figure S1 msystems.00603-25-s0001.tif The percentage relative abundance of bacteria for each Typhlocybinae sample.

Supplemental material msystems.00603-25-s0002.docx Legends for all supplemental tables, figures, and movies; Tables S2 and S3.

Graphical Abstract msystems.00603-25-s0003.jpg Graphical abstract.

Table S1 msystems.00603-25-s0004.xlsx List of samples.

Movie S1 msystems.00603-25-s0005.mp4 *M. vicina*, the 3D view of a bacteriome.

Movie S2 msystems.00603-25-s0006.mp4 *P. albicans*, the 3D view of a bacteriome.

Movie S3 msystems.00603-25-s0007.mp4 *I. stigmaticalis*, the 3D view of a fragment of a bacteriome.

Movie S4 msystems.00603-25-s0008.mp4 *P. albicans*, the 3D view of a "cap-like symbiont ball."

Movie S5 msystems.00603-25-s0009.mp4 *P. albicans*, the 3D view of a "cap-like symbiont ball."

ASM does not own the copyrights to Supplemental Material that may be linked to, or accessed through, an article. The authors have granted ASM a non-exclusive, world-wide license to publish the Supplemental Material files. Please contact the corresponding author directly for reuse.
