# Supplementary figures and images for: Extremely distinct microbial communities in closely related leafhopper subfamilies: Typhlocybinae and Eurymelinae (Cicadellidae, Hemiptera)
Source: mSystems. 2025 Jun 26;10(7):e00603-25. doi: 10.1128/msystems.00603-25 (PMC12282065; doi:10.1128/msystems.00603-25)

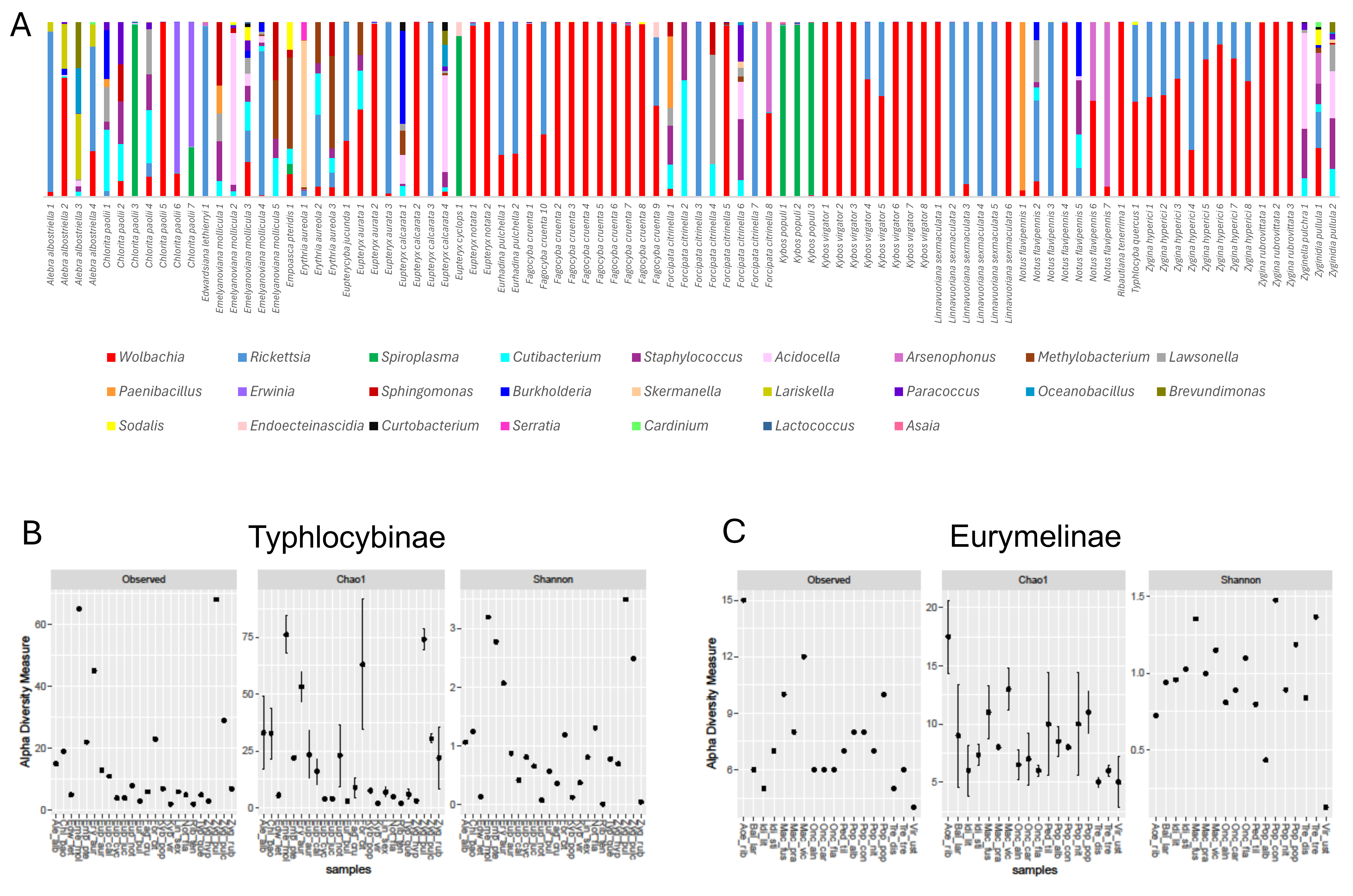

Supplement: Figure S1 — The percentage relative abundance of bacteria for each Typhlocybinae sample. [file msystems.00603-25-s0001.tif]

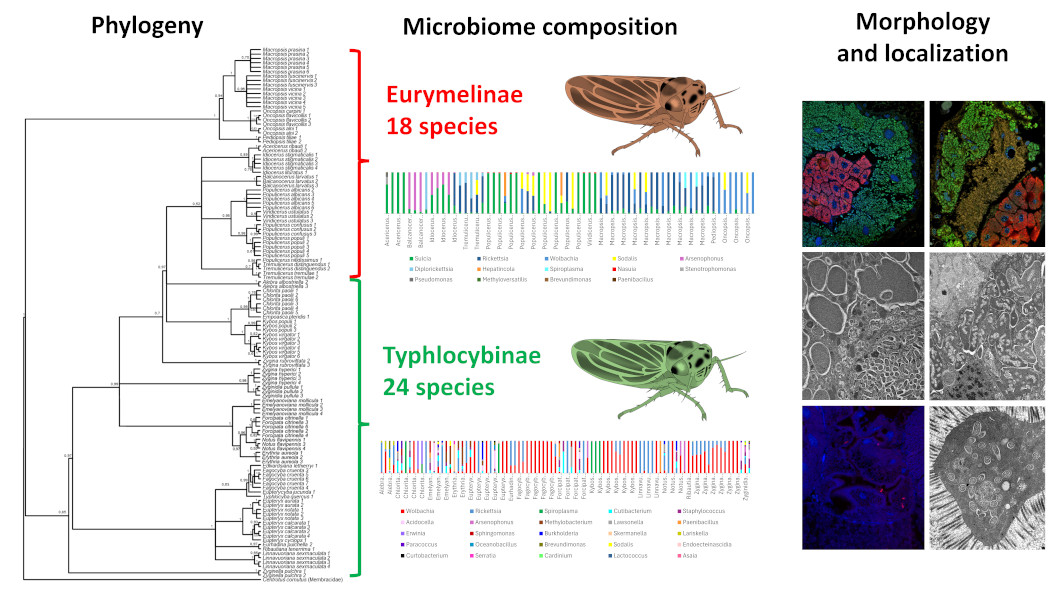

Supplement: Graphical Abstract [file msystems.00603-25-s0003.jpg]
