# Supplementary material for: Extremely distinct microbial communities in closely related leafhopper subfamilies: Typhlocybinae and Eurymelinae (Cicadellidae, Hemiptera)
Source: mSystems. 2025 Jun 26;10(7):e00603-25. doi: 10.1128/msystems.00603-25 (PMC12282065; doi:10.1128/msystems.00603-25)
Supplement: Supplemental material — Legends for all supplemental tables, figures, and movies; Tables S2 and S3. [file msystems.00603-25-s0002.docx]

**Supplementary Materials**

Table S1. All collected samples, including full names, abbreviations, place of collection, host plant, and the trophic specialty.

Table S2. Used primers, including purpose, name, sequence, target gene, annealing temperature and source.

Table S3. Results of PERMANOVA, dbRDA, generalized linear mixed-effects model (GLMM) for microbial abundance

Fig. S1. (A) The percentage relative abundance of bacteria for each Typhlocybinae sample based on the number of the sequence reads. (B) Typhlocybinae, (C) Eurymelinae, alpha diversity indices given for each species (species name abbreviations are given in Table S1).

Mov. 1. *M. vicina,* the 3D view of a bacteriome consisted of *Karelsulcia* (green) bacteriocytes and the fragment of the fat body filled within *Ophiocordyceps* yeast-like symbionts (red).

Mov. 2. *P. albicans,* the 3D view of a bacteriome consisted of two zones, formed by bacteriocytes filled with *Karelsulcia* (green) and *Nasuia* (red) bacteria.

Mov. 3. *I. stigmaticalis,* the 3D view of a fragment of a bacteriome with bacteriocytes filled with *Karelsulcia* bacteria (green) infected by *Arsenophonus* (red).

Mov. 4 and 5. *P. albicans,* the 3D view of a "cap-like symbiont ball" consisted of *Karelsulcia* (green) and *Nasuia* (red) bacteria.

Table S2. Used primers, including purpose, name, sequence, target gene, annealing temperature and source.

| **Purpose** | **Primer name** | **Primer sequence (5’-3’)** | **Target gene** | **Annealing temperature** | **Source** |
| --- | --- | --- | --- | --- | --- |
| Diagnostic PCR | F357 | CCTACGGGnGGCwGCAG | Bacterial 16S rRNA V3-V4 | 50°C | (1) |
|  | R805 | GACTAChvGGGTATCTAATCC |  |  |  |
|  | NS1 | GTAGTCATATGCTTGTCTC | Fungal 18S rRNA | 54°C | (2) |
|  | FS2 | TAGGnATTCCTCGTTGAAGA |  |  |  |
| Sequencing PCR | F357 | CCTACGGGnGGCwGCAG | Bacterial 16S rRNA V3-V4 | 50°C | (1) |
|  | R805 | GACTAChvGGGTATCTAATCC |  |  |  |
|  | Euk_ITS1F | GGTCATTTAGAGGAAGTAA | Fungal ITS1, ITS2 | 50°C | (3) |
|  | Euk_ITS4R | TCCTCCGCTTATTGATATGC |  |  |  |
|  | COIBF3_P5 | CChGAyATrGChTTyCChCG | Insects COI | 48-52°C | (4) |
|  | COIBR2_P7 | TCdGGrTGnCCrAArAAyCA |  |  |  |
| FISH | HYP760 | Cy5-CCTGCCTGGAGCACTCT | 18S rRNA gene of Ophiocordycipitaceae fungi | Not applicable | (5) |
|  | Sod1248R | Cy3-TCCGCTGACTCTCGGGAGAT | 16S rRNA gene of *Sodalis*-like symbionts | Not applicable | (6) |
|  | BET940R | Cy5-TTAATCCACATCATCCACCG | 16S rRNA gene of Betaproteobacteria | Not applicable | (7) |
|  | Sul664R | FITC-CCMCACATTCCAGYTACTCC | 16S rRNA gene of *Karelsulcia muelleri* | Not applicable | (6) |
|  | Ars2 | Cy5-TCATGACCACAACCTCCAAA | 16S rRNA gene of *Arsenophonus* symbiont | Not applicable | (8) |
|  | W1 | Rox-AATCCGGCCGArCCGACCC | 16S rRNA gene of *Wolbachia* | Not applicable | (9) |
|  | W2 | Rox-CTTCTGTGAGTACCGTCATTATC | 16S rRNA gene of *Wolbachia* | Not applicable | (9) |

**References**

1. Thijs S, De Beeck MO, Beckers B, Truyens S, Stevens V, Van Hamme JD, Weyens N, Vangronsveld J. 2017. Comparative evaluation of four bacteria-specific primer pairs for 16S rRNA gene surveys. Front Microbiol 8:251189.

2. White TJ, Bruns T, Lee S, Taylor J. 1990. Amplification and direct sequencing of fungal ribosomal RNA genes for phylogenetics. PCR Protocols 315–322.

3. GARDES M, BRUNS TD. 1993. ITS primers with enhanced specificity for basidiomycetes--application to the identification of mycorrhizae and rusts. Mol Ecol 2:113–118.

4. Elbrecht V, Braukmann TWA, Ivanova N V., Prosser SWJ, Hajibabaei M, Wright M, Zakharov E V., Hebert PDN, Steinke D. 2019. Validation of COI metabarcoding primers for terrestrial arthropods. PeerJ 2019.

5. Matsuura Y, Moriyama M, Łukasik P, Vanderpool D, Tanahashi M, Meng XY, McCutcheon JP, Fukatsu T. 2018. Recurrent symbiont recruitment from fungal parasites in cicadas. Proc Natl Acad Sci U S A 115:E5970–E5979.

6. Koga R, Bennett GM, Cryan JR, Moran NA. 2013. Evolutionary replacement of obligate symbionts in an ancient and diverse insect lineage. Environ Microbiol 15:2073–2081.

7. Demanèche S, Sanguin H, Poté J, Navarro E, Bernillon D, Mavingui P, Wildi W, Vogel TM, Simonet P. 2008. Antibiotic-resistant soil bacteria in transgenic plant fields. Proc Natl Acad Sci U S A 105:3957–3962.

8. Gottlieb Y, Ghanim M, Gueguen G, Kontsedalov S, Vavre F, Fleury F, Zchori-Fein E. 2008. Inherited intracellular ecosystem: symbiotic bacteria share bacteriocytes in whiteflies. The FASEB Journal 22:2591–2599.

9. Heddi A, Grenier AM, Khatchadourian C, Charles H, Nardon P. 1999. Four intracellular genomes direct weevil biology: Nuclear, mitochondrial, principal endosymbiont, and Wolbachia. Proc Natl Acad Sci U S A 96:6814–6819.

Table S3. Results of PERMANOVA, dbRDA, generalized linear mixed-effects model (GLMM) for microbial abundance

Results of PERMANOVA and dbRDA models for microbial abundance:

| Analysis | Model | Name | Df | SumOfSqs | F-value | R2 | p | AIC |
| --- | --- | --- | --- | --- | --- | --- | --- | --- |
| permanova | microbiome abundance ~ plant group  Blocks: insect subfamily | overall | 6 | 10,759 | 6,0498 | 0,1938 | 1,00E-04*** | -184,552 |
|  | microbiome abundance ~ plant group  Blocks: insect subfamily:genus | overall | 6 | 10,759 | 6,0498 | 0,1938 | 0,4846 | -184,552 |
|  | microbiome abundance ~ plant group  Blocks: insect genus | overall | 6 | 10,759 | 6,0498 | 0,1938 | 0,4746 | -184,552 |
|  | microbiome abundance ~ plant genus  Blocks: insect subfamily | overall | 19 | 18,482 | 3,6249 | 0,33292 | 1,00E-04*** | -183,096 |
|  | microbiome abundance ~ plant genus  Blocks: insect subfamily:genus | overall | 19 | 18,482 | 3,6249 | 0,33292 | 7,00E-04*** | -183,096 |
|  | microbiome abundance ~ plant genus  Blocks: insect genus | overall | 19 | 18,482 | 3,625 | 0,33292 | 0,0016** | -183,096 |
| dbRDA | microbiome abundance ~ plant_group + Condition(insect subfamily) | overall | 6 | 7,51 | 4,4557 | 0,151271 | 1,00E-04*** | - |
|  | microbiome abundance ~ plant_genus + Condition(insect subfamily) | overall | 19 | 14,388 | 2,9425 | 0,289812 | 1,00E-04*** | - |
|  | microbiome abundance ~ plant genus +insect genus | plant genus | 19 | 18,482 | 4,7373 | 0,332925 | 1,00E-04*** | - |
|  |  | insect genus | 15 | 11,776 | 3,8235 | 0,212127 | 1,00E-04*** | - |
|  | microbiome abundance ~ plant genus +insect subfamily | plant genus | 19 | 18,482 | 3,7797 | 0,332925 | 1,00E-04*** | - |
|  |  | insect subfamily | 1 | 1,774 | 6,8939 | 0,031956 | 1,00E-04*** | - |

Results of generalized linear mixed-effects model (GLMM) for microbial abundance:

| Model | Effect | Name | Estimate | Std. Error | z value | Variance | Std. Dev. | Chisq | Pr(>\|z\|)/Pr(>Chisq) | AIC |
| --- | --- | --- | --- | --- | --- | --- | --- | --- | --- | --- |
| Microbial abundance~plant_group+(1\|subfamily) | Fixed Effects | (Intercept) | 4,0124 | 0,1151 | 34,86 |  |  |  | <2e-16 *** | 19738.1 |
|  |  | plant_groupFagaceae | -0,85255 | 0,04382 | -19,46 |  |  |  | <2e-16*** |  |
|  |  | plant_groupHerbaceous | -0,83322 | 0,02933 | -28,41 |  |  |  | <2e-16*** |  |
|  |  | plant_groupMalvaceae | -0,32204 | 0,0631 | -5,1 |  |  |  | 3,33E-07*** |  |
|  |  | plant_groupRosaceae | 0,13979 | 0,05646 | 2,48 |  |  |  | 0,0133* |  |
|  |  | plant_groupSalicaceae | -0,17228 | 0,02853 | -6,04 |  |  |  | 1,56E-09*** |  |
|  |  | plant_groupSapindaceae | -0,76908 | 0,051 | -15,08 |  |  |  | <2e-16 *** |  |
|  | Random effect | insect subfamily |  |  |  | 0.0253 | 0.1591 |  |  |  |
|  | Overall | Intercept |  |  |  |  |  | 1215,3 | <2e-16 2*** |  |
|  |  | plant group |  |  |  |  |  | 1178,2 | <2e-16 2*** |  |
| Microbial abundance~plant_group+(1\|insect subfamily:taxa) | Fixed Effects | (Intercept) | -0,75373 | 0,67237 | -1,121 |  |  |  | 0,2623 | 14661,95 |
|  |  | plant_groupFagaceae | -0,46949 | 0,04645 | -10,108 |  |  |  | <2e-16 *** |  |
|  |  | plant_groupHerbaceous | -0,41455 | 0,03142 | -13,193 |  |  |  | <2e-16*** |  |
|  |  | plant_groupMalvaceae | -0,42479 | 0,06473 | -6,563 |  |  |  | 5,28E-11*** |  |
|  |  | plant_groupRosaceae | 0,13315 | 0,06113 | 2,178 |  |  |  | 0,0294* |  |
|  |  | plant_groupSalicaceae | -0,19616 | 0,03005 | -6,528 |  |  |  | 6,67E-11*** |  |
|  |  | plant_groupSapindaceae | 0,11185 | 0,05574 | 2,007 |  |  |  | 0,0448* |  |
|  | Random effect | insect subfamily:taxa |  |  |  | 19,26 | 4,389 |  |  |  |
|  | Overall | (Intercept) |  |  |  |  |  | 1,2567 | 0,2623 |  |
|  |  | plant_group |  |  |  |  |  | 313,8149 | <2e-16*** |  |
| Microbial abundance~plant_group+(1\|insect taxa) | Fixed Effects | (Intercept) | 2,55159 | 0,31801 | 8,024 |  |  |  | 1,03E-15*** | 15515,2 |
|  |  | plant_groupFagaceae | -0,45329 | 0,04529 | -10,008 |  |  |  | <2e-16 *** |  |
|  |  | plant_groupHerbaceous | -0,33737 | 0,02999 | -11,251 |  |  |  | <2e-16*** |  |
|  |  | plant_groupMalvaceae | -0,48153 | 0,06354 | -7,579 |  |  |  | 3,49E-14*** |  |
|  |  | plant_groupRosaceae | 0,12956 | 0,0597 | 2,17 |  |  |  | 0,03* |  |
|  |  | plant_groupSalicaceae | -0,20935 | 0,02873 | -7,286 |  |  |  | 3,19E-13*** |  |
|  |  | plant_groupSapindaceae | 0,01494 | 0,05606 | 0,267 |  |  |  | 0,79 |  |
|  | Random effect | insect taxa |  |  |  | 3,121 | 1,767 |  |  |  |
|  | Overall | (Intercept) |  |  |  |  |  | 64,378 | 1,03E-15*** |  |
|  |  | plant_group |  |  |  |  |  | 253,152 | <2e-16 2*** |  |
| Microbial abundance~plant_group+(1\|sample) | Fixed Effects | (Intercept) | 4,10225 | 0,14099 | 29,095 |  |  |  | <2e-16*** | 15412,48 |
|  |  | plant_groupFagaceae | -0,67587 | 0,24924 | -2,712 |  |  |  | 0,00669** |  |
|  |  | plant_groupHerbaceous | -0,49421 | 0,16204 | -3,05 |  |  |  | 0,00229** |  |
|  |  | plant_groupMalvaceae | -0,46879 | 0,36405 | -1,288 |  |  |  | 0,19785 |  |
|  |  | plant_groupRosaceae | -0,02183 | 0,32305 | -0,068 |  |  |  | 0,94613 |  |
|  |  | plant_groupSalicaceae | -0,26176 | 0,15764 | -1,661 |  |  |  | 0,0968, |  |
|  |  | plant_groupSapindaceae | -0,32735 | 0,29577 | -1,107 |  |  |  | 0,26839 |  |
|  | Random effect | sample |  |  |  | 0,3279 | 0,5726 |  |  |  |
|  | Overall | (Intercept) |  |  |  |  |  | 846,537 | <2e-16 *** |  |
|  |  | plant_group |  |  |  |  |  | 14,768 | 0,02214* |  |
